# Soil Microbiome Drives Depth-Specific Priming Effects in *Picea schrenkiana* Forests Following Labile Carbon Input

**DOI:** 10.3390/microorganisms13081729

**Published:** 2025-07-24

**Authors:** Kejie Yin, Lu Gong, Xinyu Ma, Xiaochen Li, Xiaonan Sun

**Affiliations:** 1 College of Ecology and Environment, Xinjiang University, Urumqi 830017, China; 2 Key Laboratory of Oasis Ecology, Xinjiang University, Urumqi 830017, China

**Keywords:** priming effect, labile carbon input, ^13^C-phospholipid fatty acids, *Picea schrenkiana*

## Abstract

The priming effect (PE), a microbially mediated process, critically regulates the balance between carbon sequestration and mineralization. This study used soils from different soil depths (0–20 cm, 20–40 cm, and 40–60 cm) under *Picea schrenkiana* forest in the Tianshan Mountains as the research object. An indoor incubation experiment was conducted by adding three concentrations (1% SOC, 2% SOC, and 3% SOC) of ^13^C-labelled glucose. We applied ^13^C isotope probe-phospholipid fatty acid (PLFA-SIP) technology to investigate the influence of readily labile organic carbon inputs on soil priming effect (PE), microbial community shifts at various depths, and the mechanisms underlying soil PE. The results indicated that the addition of ^13^C-labeled glucose accelerated the mineralization of soil organic carbon (SOC); CO_2_ emissions were highest in the 0–20 cm soil layer and decreased trend with increasing soil depth, with significant differences observed across different soil layers (*p* < 0.05). Soil depth had a positive direct effect on the cumulative priming effect (CPE); however, it showed negative indirect effects through physico-chemical properties and microbial biomass. The CPE of the 0–20 cm soil layer was significantly positively correlated with ^13^C-Gram-positive bacteria, ^13^C-Gram-negative bacteria, and ^13^C-actinomycetes. The CPE of the 20–40 cm and 40–60 cm soil layers exhibited a significant positive correlation with cumulative mineralization (CM) and microbial biomass carbon (MBC). Glucose addition had the largest and most significant positive effect on the CPE. Glucose addition positively affected PLFAs and particularly microbial biomass. This study provides valuable insights into the dynamics of soil carbon pools at varying depths following glucose application, advancing the understanding of forest soil carbon sequestration.

## 1. Introduction

Soil organic carbon (SOC) stocks in forest ecosystems are primarily influenced by organic matter inputs from plant litter and the carbon released during SOC decomposition [1]. With global warming, the decomposition, transformation, and accumulation processes of plant litter and root exudates in soil within forest ecosystems are affected [2]. Rising temperatures drive forest ecosystems to produce more readily decomposable organic carbon input by altering the total amount of photosynthetic carbon fixation and its allocation strategy (e.g., shifting towards litter production and root exudation). Increased plant litter production and enhanced root metabolic activity promote the allocation of more photosynthetic carbon belowground by plants, significantly increasing the exudation fluxes and chemical availability of root exudates (such as sugars and organic acids) [3,4]. The input of exogenous organic matter can either enhance or inhibit the SOC mineralization dynamics of native soil organic carbon (SOC), thereby generating positive or negative soil priming effects [5].

The priming effect (PE) is a potential mediator of soil carbon balance by integrating soil carbon inputs and outputs, and it represents a key mechanism regulating soil carbon dynamics [6]. The amount of carbon input affects the direction and magnitude of the priming effect. However, the sensitivity of the PE to carbon inputs exhibits variability depending on soil properties and carbon addition approaches [7]. In subtropical broad-leaved evergreen forests, glucose addition was found to induce a higher PE at lower input levels, whereas the strength of the PE was instead lower when glucose input was higher compared to lower inputs [8]. Research findings are inconsistent regarding the changes in the intensity and direction of soil PE induced by inputs of readily labile organic matter [9,10].

The intensity and direction of the priming effect are influenced by soil physical and chemical properties, nutrient availability, and the composition of the soil microbial community at varying depths [11,12,13,14]. Studies have demonstrated that the intensity of the PE is negatively correlated with the SOC content [15,16]. Changes in SOC content are often associated with alterations in soil texture, porosity, and other physicochemical properties, which collectively affect microbial decomposition processes and consequently the soil PE [17]. Soil pH can alter the physicochemical protection of SOM and directly modulate the PE through abiotic mechanisms. In acidic soils, aluminum ions complex with carboxyl groups of organic matter (SOM), altering adsorption, weakening physical protection, and facilitating microbial decomposition of SOM, thus increasing PE. Concurrently, iron and manganese oxides, whose solubility increases in acidic soils, affect the direction and intensity of the PE by undergoing redox reactions with SOM [18]. Soil nitrogen and phosphorus availability significantly regulates PE intensity and direction by modulating microbial metabolic efficiency and nutrient stoichiometry. Phosphorus addition may result in a relative decrease in nitrogen availability, leading to an increased intensity of the PE through microbial mining of nitrogen from soil organic matter [19].

Most mechanisms underlying the PE involve microorganisms, with changes in microbial biomass and community composition directly influencing soil organic matter decomposition and the PE. Nutrient limitation plays a decisive role in microbial activity; when added nutrients satisfy microbial requirements, the new substrate provides additional nutrients for microbial growth, leading to shifts in microbial assimilation or life strategies [20,21] and enhancing microbial decomposition capacity [22]. Most current studies indicate that when glucose is introduced into the soil, microbial community succession occurs alongside decomposition [23,24]. In the early stages of labile organic matter addition, bacterial growth is predominantly stimulated, while fungi become more dominant as the labile organic matter is gradually consumed, resulting in a significant increase in the fungi-to-bacteria ratio [25,26].

Prior studies have revealed carbon reserves in deeper soil layers exhibit greater stability compared to those near the surface [27,28]. With increasing soil depth, nutrient concentrations in deeper layers decline relative to surface soils. Nutrient availability limits the decomposition of organic matter in deep soils [29,30,31]. Investigations have revealed that differences in the composition and functionality of microbial communities between soil strata separated by a vertical distance of 10 cm are comparable to those observed among communities spanning thousands of kilometers at the surface level [32]. Moreover, microbial activity decreases with soil depth as a consequence of variations in nutrient content and hydrothermal and aeration conditions, consequently influencing how soils at various depths respond to external carbon inputs. While the PE has been extensively studied in different soil carbon pools [33,34], additional research is necessary to clarify the responses of deeper soils within Tianshan forest ecosystems to labile external carbon inputs and the resultant alterations in microbial community structures during the soil PE.

*Picea schrenkiana*, as the dominant tree species occupying over 90% of forestland in the Tianshan Mountains, plays critical roles in ecosystem services, including soil conservation, biodiversity maintenance, and timber accumulation. Previous studies on the Tianshan forest ecosystem have primarily focused on large-scale carbon storage [35], forest productivity [36], and vegetation spatial distribution [37,38], while research on the priming effects of exogenous carbon inputs on forest floor soil organic carbon and microbial communities under controlled laboratory conditions remains largely unexplored. This study utilized ^13^C-labeled glucose a source of as labile carbon, conducting an indoor incubation experiment that incorporated stable isotope tracer techniques and bioinformatics analyses. The aim was to uncover the molecular-level response mechanisms of microbial communities to carbon inputs and to assess how varying carbon additions influence the PE of SOC in forest ecosystems and its dynamic process. We hypothesized that (1) the soil response to additional carbon inputs will diminish when a certain threshold of readily glucose inputs is reached and that this response will be inconsistent at different soil depths and that (2) glucose inputs have time-dynamic variations in the effects of SOC mineralization and PE by altering microbial communities at different soil depths. Examining the mechanisms by which glucose additions impact the PE across different soil depths and analyzing the subsequent changes in soil main microbial groups can enhance our understanding of SOC and contribute to promoting forest soil C sequestration science.

## 2. Materials and Methods

### 2.1. Study Location and Soil Sample Collection

The research site is situated in the *Picea schrenkiana* forest region (43°29′ E, 87°11′ N) near Nanshan Observatory in Urumqi County, situated within the mid-mountain belt on the northern slope of the Tianshan Mountain. *Picea schrenkiana* is the dominant and extensively distributed forest species in this region. The area exhibits a temperate continental climate, characterized by an average annual temperature ranging between 0 °C and 4 °C and significant temperature fluctuations throughout the year. The soil is classified as gray-brown forest soil, featuring a thick humus layer and slight acidity. The soil type is chernozem (USDA Soil Taxonomy). In September 2023, three sampling plots with flat topography and similar slope orientations were selected within the *Picea schrenkiana* forest. In this study, three research plots of 10 m × 10 m were set up, and each plot was spaced more than 50 m apart to eliminate spatial autocorrelation and ensure the independence between the plots. Soil samples were collected using a five-point sampling method at 0–20 cm, 20–40 cm, and 40–60 cm depths with a soil auger. Samples from identical depths across five points within each plot were homogenized and root fragments removed. Samples were transported to the laboratory in sterile and sealed bags. A portion of the soil was dried, passed through a 2 mm sieve, and used to analyze soil physical and chemical properties, organic matter composition, and conduct subsequent experiments. Fresh soil samples were stored at −80 °C for determining soil microbial biomass C (MBC), microbial biomass nitrogen (MBN), and PLFAs.

### 2.2. Incubation Experiment Design and CO_2_ Analysis

Before initiating the incubation experiment, physicochemical parameters such as soil organic carbon (SOC), total nitrogen (TN), total phosphorus (TP), soil water content (SWC), and bulk density were measured (Table S1). In the experiment, 50 g of air-dried soil was transferred into 250 mL pre-labeled culture flasks, with soil moisture content subsequently modified to 50% of saturated field capacity. The soil was pre-incubated for one week in the dark at 20 °C to allow microbial activity to recover. After pre-incubation, a glucose solution containing ^13^C-labeled glucose (99 atom%; American CIL) and unlabeled glucose mixed in a 1:25 ratio was added. The diluted glucose solution had a ^13^C abundance of 5 atom% (*δ*^13^C = 4083‰).Three concentrations of ^13^C-glucose were established by referring to prior experimental designs involving glucose addition and SOC content [8,33,39,40]. Glucose solutions representing 1% SOC (LA), 2% SOC (MA), and 3% SOC (HA) for different soil depths were applied to 50 g of soil (Table S2). A control treatment (CK) without ^13^C-labeled glucose was included (adding an equal amount of deionized water). The total number of samples is 3 sites × 3 soil layers × 4 treatments (CK, LA, MA, and HA) × 5 replicates. Deionized water was used to adjust all soil samples to 65% of saturated field capacity, and the samples were then incubated for 52 days under the same conditions as the pre-incubation. Additionally, three control flasks without soil were set up to assess the baseline CO_2_ production. The static alkali absorption technique was employed to quantify CO_2_ released during incubation, using NaOH. The CO_2_-C content in the 1 M NaOH (15 mL) was determined by titration, allowing calculation of the release rate and cumulative CO_2_ output. On days 1, 3, 7, 12, 19, 28, 38, and 52, the NaOH solution was replaced. To precipitate absorbed CO_2_, 0.5 mol/L BaCl_2_ was introduced into the solution, and the remaining NaOH was titrated with 0.5 mol/L HCl. The precipitate was centrifuged at 1200 r/min three times, the supernatant was removed, and the precipitate was then dried.

The δ^13^C isotope composition of the BaCO_3_ precipitate was analyzed using an isotope ratio mass spectrometer, and these measurements were used to quantify the CO_2_ emissions originating from glucose. At the end of incubation period, soil was destructively sampled to measure MBC, MBN, PLFAs, and other physical and chemical properties. Throughout the incubation period, the SWC was monitored using the weighing method, with deionized water added as needed to maintain consistent moisture levels. Soil incubation was carried out using strict sealing to prevent any gas leakage.

### 2.3. Determination of Soil Physicochemical Properties and Analysis of PLFAs

Soil pH and EC were evaluated using a potentiometric method [41]. The Walkley–Black oxidation method (K_2_Cr_2_O_7_-H_2_SO_4_) was employed for SOC quantification. Total nitrogen content was measured by the Kjeldahl method, while total phosphorus was determined using the Mo-Sb colorimetric technique following digestion with HClO_4_-H_2_SO_4_ (Table S3). MBC and MBN were determined by chloroform extraction fumigation [42]. The composition of main soil microbial groups was determined by PLFA analysis

Soil PLFA analysis was employed to characterize the microbial community structure [43,44]. Extracts underwent both quantitative and qualitative analyses using micro GC-C-IRMS, which is equipped with a combustion column and coupled with GC combustion III and Delta V Advantage isotope ratio mass spectrometry for measuring the ^13^C/^12^C ratios of individual PLFA [45,46]. The following PLFA markers were used to identify specific microbial groups: i15: 0, i16: 0, i17: 0, a15: 0, and a17: 0 represent Gram-positive bacteria (G^+^); 16: 1ω5c, 16: 1ω7c, 18: 1ω7c, and cy17: 0 denote Gram-negative bacteria (G^−^); 10Me16: 0, 10Me17: 0, and 10Me18: 0 are indicative of actinomycetes; 18: 1ω9c and 20: 1ω9c correspond to fungi; 14: 00, 16: 00, and 18: 00 are non-specific (universal) PLFA markers [24,47].

### 2.4. Index Calculation

The two-source mixing model was employed to differentiate the contributions of SOC decomposition (C_S_) and glucose-derived carbon (C_G_) to total soil respiration at each sampling point. The model is expressed as follows [8]:

(1) $$\;C_{S}=\frac{C_{T}(\delta_{G}{-\delta }_{T})}{\delta_{G}{-\delta }_{S}}$$

(2) $$C_{G}=C_{T}-C_{S}$$

where C_T_, C_G_, and C_S_ denote, respectively, the total soil carbon content (i.e., total carbon in the lye solution), the glucose-derived carbon content (i.e., CO_2_-C from glucose decomposition), and the soil organic matter-derived carbon content (i.e., CO_2_-C from SOC mineralization), expressed in mg C kg^−1^ soil day^−1^; *δ*_T_, *δ*_G_, and *δ*_S_ represent, respectively, the *δ*^13^C values of total soil carbon in the glucose-added treatments, the *δ*^13^C values in the glucose solution (across three levels), and the *δ*^13^C values of SOC in the control treatment (without glucose addition).

Primed C represents the absolute priming effect (mg C kg^−1^), defined as the difference between the CO_2_-C derived from soil organic carbon decomposition in the soil total respiration (C_S_) and that from the control treatment (C_C,_ i.e., without glucose addition):

(3) $$Primed\;C=C_{S}-C_{C}$$

Cumulative PE (mg C kg^−1^) denotes the difference between the cumulative CO_2_-C release from SOC decomposition in the glucose addition treatment (C_S_) and that in the control treatment (Cc) over a 52-day period. This period is subdivided into n intervals, and for each interval, the mean absolute PE, calculated as the average of the initial (P_0_) and final (P_t_) values, is multiplied by the interval duration (t) to yield the PE for that interval. The total PE is then the sum of the PE values for all intervals (t_1_, t_2_, t_3_, …).

(4) $$Cumulative\;PE=\sum_{1}^{n}\left(\frac{P_{0}+P_{t}}{2}\right)\;(n\;=\;1,\;2\;\ldots \;8)$$

The *δ*^13^C value of each phospholipid fatty acid molecule was corrected by the equilibrium equation to remove the effect of C introduced by methyl esterification:

(5) $$\;\delta^{13}C_{PLFA}=\frac{(n+1)\delta^{13}C_{FAME}-\delta^{13}C_{MeOH}}{n}$$

In this equation, n represents the number of C atoms, and *δ*^13^C_PLFA_ represents the *δ*^13^C of the PLFA monomer. *δ*^13^C_FAME_ and *δ*^13^C_MEOH_ represent a single fatty acid methyl ester and *δ*^13^C used for methyl esterification. The amounts of ^13^C labeled and unlabeled PLFAs were used to represent microbial biomass using glucose and soil organic matter, respectively [48].

(6) $$\;\delta^{13}C_{PLFA}=\frac{(n+1)\delta^{13}C_{FAME}-\delta^{13}C_{MeOH}}{n}$$

In this formula, PLFA_G_ represents the amount of microbial PLFAs using glucose and soil organic matter. The *δ*^13^C_G0_ and *δ*^13^C_Gi_ represent the *δ*^13^C abundance values of PLFAs without and with glucose treatment, respectively.

### 2.5. Statistical Analysis

Statistical analyses were performed using R version 3.4.3. Prior to significance testing, the Levene test was conducted to verify data normality and homogeneity of variances. ANOVA was applied to compare the ^13^C-labeled microbial PLFA groups and overall PLFAs among different carbon input treatments. In addition, two-way ANOVA evaluated the effects of glucose input, soil depth, and their interaction on soil microbial biomass carbon and nitrogen. Pearson correlation analysis was then used to examine the relationships between the ^13^C-PLFA microbial groups and several parameters, including cumulative SOC, cumulative PE, soil physicochemical properties, MBC, and MBN. A PLS-PM was constructed using the “plspm” package (version 0.4.9) to assess the direct and indirect effects of decomposed carbon input, soil depth, soil physicochemical properties, microbial biomass, and PLFAs on PE and to identify the primary driving factors of PE. In model construction, microbial biomass was quantified based on the measured carbon and nitrogen content of soil microorganisms, whereas soil physicochemical properties comprised soil pH, conductivity, organic carbon, total nitrogen, and total phosphorus. Different types of PLFAs were treated as observed variables and linked to the latent variable PLFAs through the external model. To mitigate multicollinearity, the VIF for all variables was maintained below 10. External model loading values were set above 0.7 to exclude independent variables with minimal contributions. Finally, path coefficients and certainty coefficient estimates were validated using a self-guided method (1000 iterations), and the overall predictive performance of the model was assessed via the goodness-of-fit index.

## 3. Results

### 3.1. Soil Organic Carbon Mineralization

**Figure 1:**
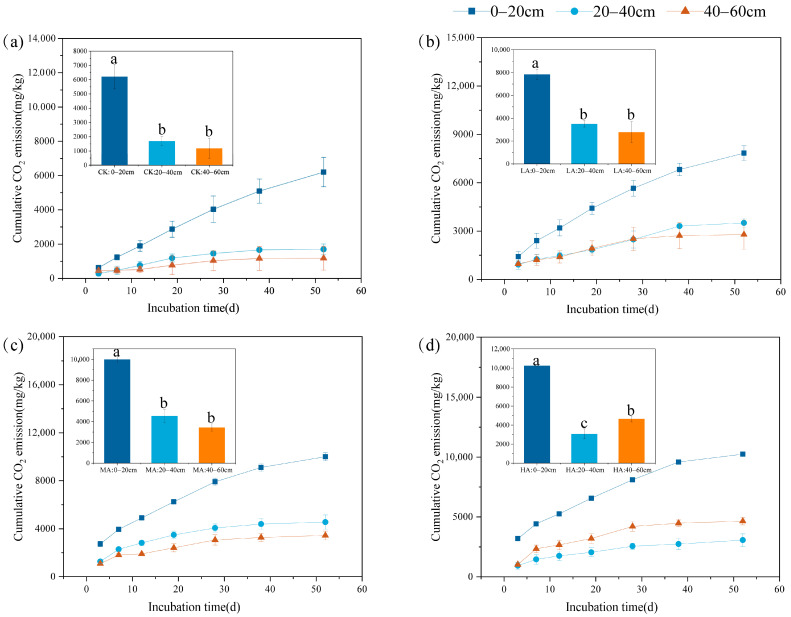
Effects of different glucose additions on total CO_2_ emissions in different soil layers. (**a**) Control treatment (CK); (**b**) 1% SOC glucose-added treatment (LA); (**c**) 2% SOC glucose-added treatment (MA); (**d**) 3% SOC glucose-added treatment (HA). The histogram shows the cumulative CO_2_ emissions from the soil layers at the end of the 52-day incubation. Lowercase letters indicate significant differences in cumulative carbon dioxide emissions between different soil layers (*p* < 0.05).

Under different glucose addition treatments, the 0–20 cm soil layer exhibited the highest cumulative CO_2_ emissions at each sampling point and was significantly higher than other soil layers (*p* < 0.05) (Figure 1). CO_2_ emissions decreased with increasing soil depth but were higher in the 40–60 cm layer than in the 20–40 cm layer under the HA treatment. Over time, CO_2_ emissions initially increased rapidly and then stabilized. At the end of the culture, the values were, respectively, 7838.6 ± 451.13, 10,001.2 ± 342.24, 10,238.8 ± 24.89, and 6215 ± 861.82 mg C kg^−1^.

**Figure 2:**
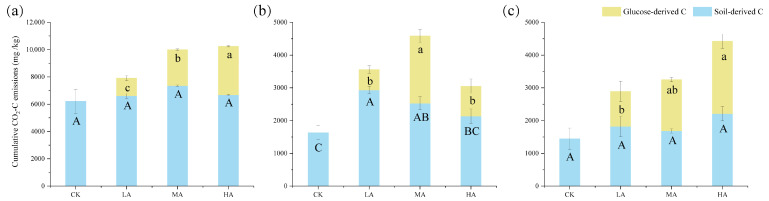
Cumulative CO_2_ emissions from soil and glucose sources. At the end of 52 days of incubation, the cumulative carbon dioxide emissions from glucose and soil organic matter sources in the (**a**) 0–20 cm; (**b**) 20–40 cm; (**c**) 40–60 cm soil layers. Note: CK: control treatment; LA: 1% SOC glucose-added treatment; MA: 2% SOC glucose-added treatment; HA: 3% SOC glucose-added treatment. Capital letters indicate significant differences in cumulative carbon dioxide emissions derived from soil organic matter (SOM) among different treatments (*p* < 0.05); lowercase letters indicate significant differences in cumulative carbon dioxide emissions derived from glucose among different treatments (*p* < 0.05).

Glucose input stimulated the emissions of CO_2_ from SOC. Across different soil layers, both glucose-derived CO_2_ emissions and total CO_2_ emissions increased significantly with the addition of glucose (Figure 2) (*p* < 0.05). However, in the 20–40 cm soil layer, both the CO_2_ emissions from glucose sources and the total CO_2_ emissions first increased and then decreased significantly. Moreover, the increase in glucose addition led to a significant reduction in the emissions of original soil organic carbon (*p* < 0.05).

### 3.2. The Priming Effect of Soil Organic Carbon

**Figure 3:**
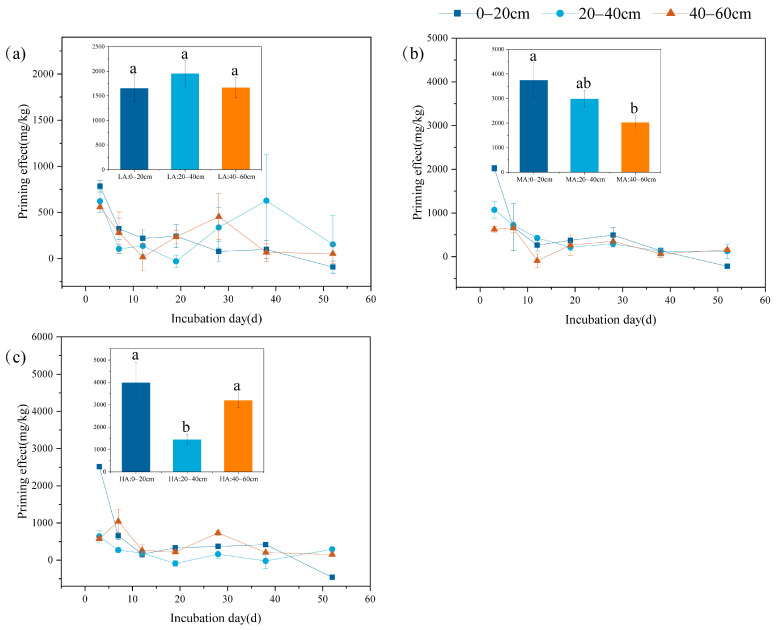
Effects of glucose addition on soil PE in different soil layers. (**a**) 1% SOC glucose-added treatment (LA); (**b**) 2% SOC glucose-added treatment (MA); (**c**) 3% SOC glucose-added treatment (HA). The histogram shows the cumulative soil PE from different soil layers at the end of 52 days of incubation. Lowercase letters indicate significant differences in the priming effect between different soil layers (*p* < 0.05).

PE exhibited pronounced spatial heterogeneity across different soil layers. Under the LA treatment, the 20–40 cm soil layer demonstrated the highest PE intensity, while the 40–60 cm subsoil showed the lowest cumulative PE. Conversely, under HA treatment, the surface soil displayed maximum PE values, followed by the 40–60 cm layer, with the 20–40 cm layer exhibiting minimal cumulative PE. While glucose addition stimulated glucose-derived CO_2_ emissions, suppressed organic carbon mineralization leading to negative PE was observed during incubation (Figure 3). Surface soils consistently manifested negative PE across all treatments by the experiment’s conclusion. In LA-treated 20–40 cm soils, transient negative PE (−29.33 ± 67.21 mg C kg^−1^) emerged at day 19 of incubation, followed by a progressive shift to positive PE, peaking at 337.33 ± 221.46 mg C kg^−1^ by day 28 and subsequently declining thereafter.

### 3.3. Soil Microbial Communities

**Figure 4:**
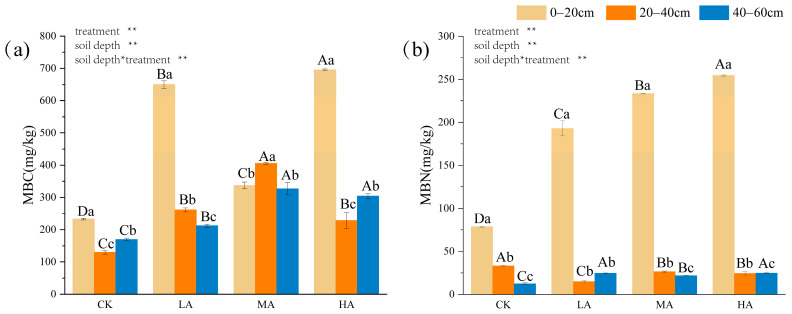
Effects of glucose addition on soil microbial carbon and nitrogen content in different soil layers. (**a**) MBC content (mg/kg); (**b**) MBN content (mg/kg). The significant differences between treatments and soil depths are indicated by different lowercase and uppercase letters, respectively. Significant differences (*p* < 0.05) between different soil layers are indicated by capital letters; significant differences (*p* < 0.05) between different treatments are indicated by lower case letters. The results of the two-way ANOVA are shown in the upper left corner of the graph, with * denoting significant and ** denoting highly significant. Note: CK: control treatment; LA: 1% SOC glucose-added treatment; MA: 2% SOC glucose-added treatment; HA: 3% SOC glucose-added treatment.

Significant differences (*p* < 0.05) were observed in MBC and MBN content between treatments at different soil depths (Figure 4). The responses of MBC and MBN to carbon inputs varied across soil layers. In the 20–40 cm and 40–60 cm layers, MBC initially increased with rising carbon inputs and subsequently decreased, whereas in the 0–20 cm layer, MBC increased with higher carbon inputs but was suppressed under the MA treatment. MBN displayed a similar trend across treatments and soil depths, with the highest MBN content observed in the 0–20 cm layer, where it increased significantly (*p* < 0.05) with rising carbon inputs.

**Figure 5:**
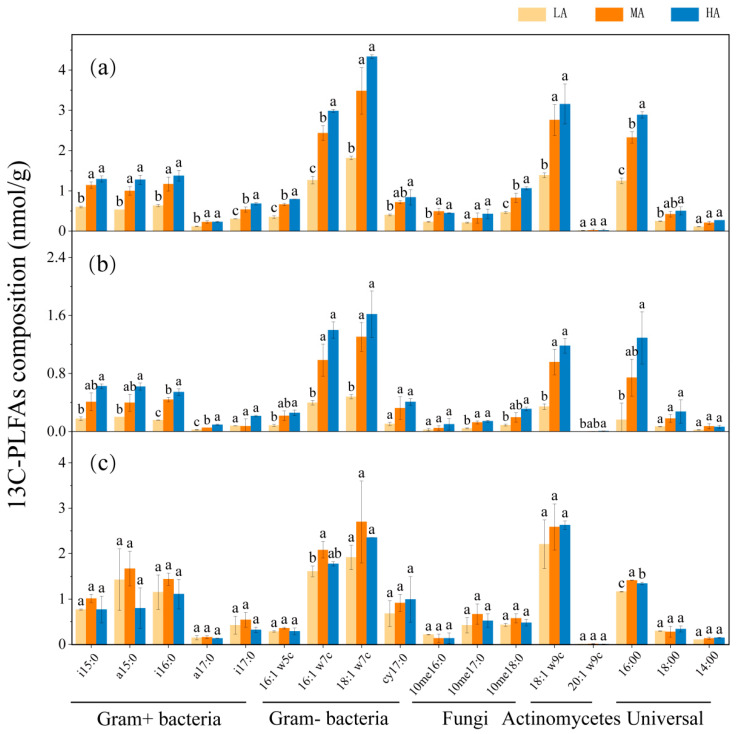
Effects of glucose addition on microbial ^13^C-PLFAs in different soil layers: (**a**) 0–20 cm; (**b**) 20–40 cm; (**c**) 40–60 cm. The significant difference between different treatments (*p* < 0.05) is expressed in different lowercase letters. Note: LA: 1% SOC glucose-added treatment; MA: 2% SOC glucose-added treatment; HA: 3% SOC glucose-added treatment.

Glucose-derived carbon was predominantly assimilated into 16: 1w7c and 18: 1w7c, with subsequent incorporation into 18:1 ω9c, i15: 0, a15: 0, and i16: 0 after a 52-day incubation period (Figure 5). Significant increases in the monomer concentrations of most ^13^C-PLFAs were observed in both 0–20 cm and 20–40 cm soil layers upon glucose amendment (*p* < 0.05). In the 0–20 cm and 20–40 cm soil layers, the monomer content of most ^13^C-PLFAs increased significantly with glucose addition (*p* < 0.05). The trends in the monomer contents of different ^13^C-PLFAs with increasing glucose inputs were similar, with ^13^C-PLFAs content in the 0–20 cm layer being higher than that in the other two soil layers. The absolute contents of ^13^C-PLFAs i15: 0, i16: 0, i17: 0, a15: 0, and a17: 0 (Gram-positive) were higher in the 40–60 cm soil layer than in the 0–20 cm and 20–40 cm layers.

**Figure 6:**
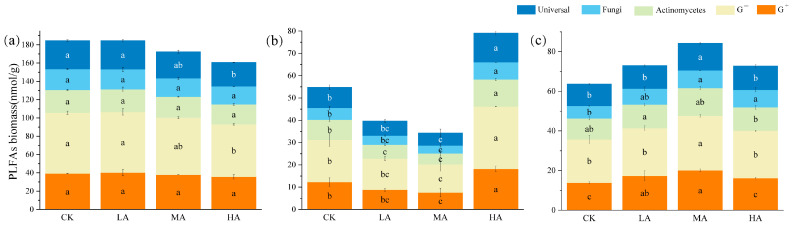
Effects of glucose addition on microbial PLFAs in different soil layers: (**a**) 0–20 cm; (**b**) 20–40 cm; (**c**) 40–60 cm. The significant difference between different treatments (*p* < 0.05) is expressed in different lowercase letters. Note: CK: control treatment; LA: 1% SOC glucose-added treatment; MA: 2% SOC glucose-added treatment; HA: 3% SOC glucose-added treatment.

In the 0–20 cm soil layer, PLFAs, bacterial biomarkers (including Gram-positive and Gram-negative bacteria) as well as fungal and actinomycete biomarkers were significantly higher than in the 20–40 cm and 40–60 cm layers (Figure 6). In the 0–20 cm soil layer, the accumulation of PLFA content was inhibited with increasing carbon input. Microbial biomass in the 20–40 cm layer increased significantly as glucose addition decreased, with similar response trends observed across various microbial groups. In the 40–60 cm soil layer, the content of total PLFAs, common bacteria, Gram-positive bacteria, and Gram-negative bacteria under MA treatment was significantly higher than in the other treatments.

### 3.4. Driving Factors Affecting the PE of Labile Carbon Inputs on Soil Organic Carbon

**Figure 7:**
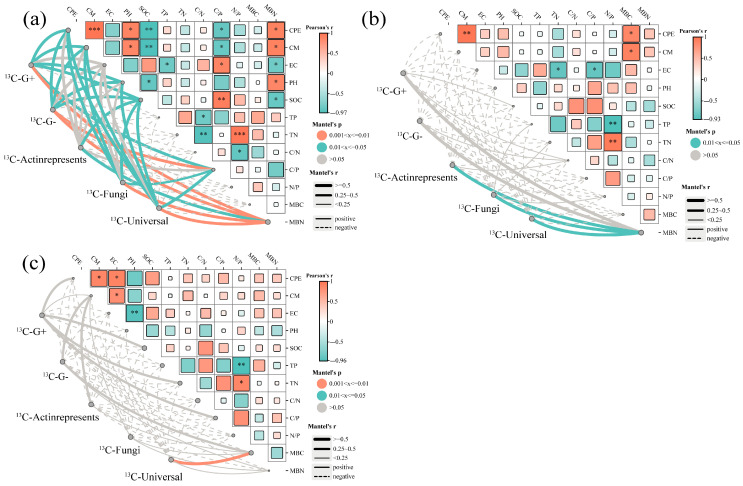
The Pearson’s correlation between the accumulated stimulation effect, SOC mineralization, and soil physical and chemical properties of various microbial groups of ^13^C-phospholipid fat acids in soil at 0–20 cm (**a**), 20–40 cm (**b**), and 40–60 cm (**c**) with glucose addition. ^13^C-G^+^ represents the ^13^C-gram positive bacteria community; ^13^C-G^−^ represents the ^13^C-gram negative bacteria community; ^13^C-actinomycetes represents the ^13^C-actinomycetes community; ^13^C-Fungi represents the ^13^C-fungi community; ^13^C-Universal represents the ^13^C-non-specific bacteria community; CPE represents the accumulated stimulation effect; CM represents the accumulated mineralization. Edaphic variables include EC (electrical conductivity), PH (pH value), SOC (soil organic carbon), TN (total nitrogen), TP (total phosphorus), C/N ratio, N/P ratio, MBC (microbial biomass carbon), and MBN (microbial biomass nitrogen). Network edges encode correlation strength (line width) and directionality (solid = positive correlations; dashed = negative correlations), with significance levels denoted by * *p* < 0.05, ** *p* < 0.01 and *** *p* < 0.001.

In the 0–20 cm soil layer, CPE was positively correlated with ^13^C-G^+^ (Gram-positive bacteria), ^13^C-G^−^, and ^13^C-actinomycetes (*p* < 0.05) (Figure 7). The changes of ^13^C-G^+^, ^13^C-G^−^, ^13^C-actinomycetes, and ^13^C-Universal were significantly correlated with CM and positively correlated with MBN (*p* < 0.05). In the 40–60 cm soil layer, ^13^C-Universal was significantly and positively correlated with MBC. In the 0–20 cm soil layer, CPE and CM were significantly and positively correlated with MBN and pH and significantly and negatively correlated with SOC and C/P (*p* < 0.05). In the 20–40 cm and 40–60 cm soil layers, CPE and CM were significantly and positively correlated with MBN (*p* < 0.05).

**Figure 8:**
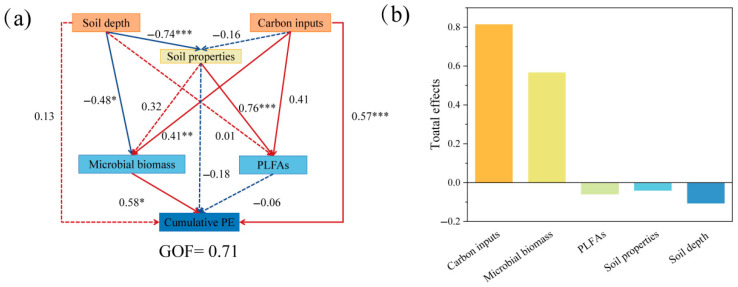
(**a**) Partial least squares path modeling (PLS-PM) was used to analyze the possible pathways affecting the CPE of soil. Red and blue arrows indicate positive and negative causality, respectively; numbers on the arrows indicate normalized path coefficients. Dashed lines indicate nonsignificant, and solid lines are significant. (**b**) The total effect of different factors on the influence of soil PE, with significance levels denoted by * *p* < 0.05, ** *p* < 0.01, and *** *p* < 0.001. GOF: goodness of fit, which indicates the reliability of the structural equation model.

PE was positively regulated by glucose inputs and microbial biomass and negatively regulated by soil depth, soil properties, and PLFAs (GOF = 0.71) (Figure 8). Glucose input had the largest and most significant positive effect on CPE (path coefficient = 0.57). Soil depth and glucose addition had direct positive effects on SOC stimulation and affected SOC stimulation by influencing soil physicochemical properties, microbial biomass, and PLFAs. Among these, soil depth changes had a significant negative effect (path coefficients of 0.74 and 0.48) on soil properties and microbial biomass, while glucose addition positively affected PLFAs and microbial biomass, which in turn had a significant effect on microbial mass (path coefficient = 0.41). Soil properties had significant positive effects on PLFAs (path coefficient = 0.76). Soil properties and PLFAs had negative effects on PE.

## 4. Discussion

### 4.1. Effects of Soil Depth and Glucose Input Amount on Organic Carbon Mineralization and Priming Effect

SOC mineralization is influenced by several factors, including chemical and physical protection, soil nutrient content, and microbial community structure [14,28]. Soil depth emerges as a critical determinant of both CO_2_ emission dynamics and microbial metabolic activity [49]. In this study, CO_2_ emissions from the 0–20 cm soil layer were consistently higher than those from the 20–40 cm and 40–60 cm soil layers and showed a decreasing trend with increasing soil depth (Figure 1), which is consistent with findings from other studies in the field [50]. In deeper soils, microbial activity is often limited by factors such as substrate availability, leading to lower CO_2_ emissions [51]. Surface soils have higher carbon mineralization rates, more active microbial communities, and more significant responses to exogenous carbon sources [52]. Under certain conditions, increased glucose addition may lead to CO_2_ emissions from the 40–60 cm soil layers that exceed those from 20–40 cm soils layers (Figure 2 and Figure 3). Existing studies have shown that glucose addition provides a readily available carbon source that supplies energy to microorganisms and partially alleviates limitations imposed by nutrient deficiencies [53]. Moreover, indoor-cultivated soils, through sieving and other disturbances, can alter the physicochemical properties of deeper soils, making some organic matter that was previously protected both physically and chemically more accessible and usable by microorganisms [54]. Consequently, CO_2_ emissions in deeper soils surpass those in shallow soils [12,55].

The impact of soil depth on the soil organic carbon (SOC) priming effect involves complex mechanisms. In this study, although soil depth exerted a direct positive influence on the priming effect, its overall effect was inhibitory due to the negative indirect impacts mediated through soil physicochemical properties and microbial biomass (with path coefficients of 0.74 and 0.48, respectively) (Figure 8). A large-scale transect study conducted in the grasslands of Inner Mongolia revealed that the intensity of the priming effect decreased with increasing soil depth, and the surface soil exhibited a positive priming effect, while the middle and deep soil layers showed negative priming effects. The priming effect in deep soils was primarily controlled by soil substrates [56]. Despite the discrepancy in the direction of the priming effect response to soil depth between that study and the present research, both emphasized the significance of soil depth in studies on the priming effect. Specifically, increasing soil depth leads to the deterioration of soil physicochemical properties (such as nutrient loss), thereby inhibiting the SOC priming effect. Meanwhile, the reduction in soil microbial biomass weakens the role of microorganisms in the decomposition and transformation of organic carbon, further decreasing the intensity of the priming effect.

A non-linear relationship was observed between exogenous carbon input and SOC mineralization [57,58]. While glucose input initially stimulated the mineralization of original SOC, this stimulation diminished as glucose input increased. The 20–40 cm soil layer exhibited a lower reduction in original SOC emissions under the MA treatment compared to the LA treatment, with similar responses observed in other soil layers, which aligned with our hypothesis. However, not all soil layers in this study showed a single-peak pattern of initial increase followed by a decrease. As a readily available carbon source, glucose activates microbial metabolic activities, allowing microorganisms to utilize glucose while also enhancing the decomposition and utilization of original SOC, thereby stimulating the emission of the latter. Nevertheless, with increasing glucose input, microbial growth may enter a phase of carbon excess, where microorganisms preferentially utilize the abundant glucose [24]. At the same time, feedback regulatory mechanisms may inhibit the secretion or activity of enzymes associated with the decomposition of original SOC, thereby reducing the decomposition of the original organic carbon [59].

Studies have indicated that the addition of exogenous carbon sources can temporarily inhibit SOC mineralization; however, this inhibitory effect is typically short-lived, and SOC mineralization may recover or even increase over time. This suggests that the effect of exogenous carbon addition on the soil carbon cycle is time-dependent [14,57,60]. Similar dynamics were observed in our study, where glucose addition increased CO_2_ emissions from the soil while also triggering a negative PE. This effect was observed in all soil layers in this study, but it manifested differently across soil layers and glucose concentrations (Figure 3). Under the LA treatment, the 20–40 cm soil layer exhibited a negative PE (−29.33 ± 67.21 mg C kg^−1^) at 19 days of incubation, but this effect gradually shifted from negative to positive over time, reaching a significant positive PE at 28 days (337.33 ± 221.46 mg C kg^−1^) (Figure 3). This may be associated with the preferential use of exogenous carbon sources by microbial communities. Previous research has shown that microorganisms typically reduce the decomposition of refractory organic matter when abundant and readily available carbon sources are present [25]. This phenomenon is linked to a decrease in SOC mineralization. However, as exogenous carbon sources are gradually depleted, microorganisms may turn to SOC, resulting in a positive PE in the subsequent periods. Furthermore, the influence of varying glucose concentrations on negative PE yielded different outcomes. The negative PE was more pronounced under the HA treatment. For instance, in the 20–40 cm soil layer, the negative PE was observed at 19 and 38 days of incubation, with values of −88.00 ± 76.21 and −22.00 ± 202.23 mg C kg^−1^, respectively (Figure 3). The addition of a high concentration of glucose may inhibit SOC mineralization to a greater extent, further confirming the complex effects of exogenous carbon sources on soil carbon dynamics. Results from incubation experiments on the priming effect across different soil layers in subtropical Moso bamboo forests also indicated that the impact of biochar is depth- and time-dependent. During the early incubation stage, biochar inhibited organic carbon mineralization, resulting in a negative priming effect, with the lower soil layer (20–40 cm) exhibiting a more pronounced negative priming effect. However, this shifted to a positive priming effect over time, further confirming the complex influence of exogenous carbon sources on soil carbon dynamics [61].

### 4.2. Microbial and Abiotic Drivers of Soil Organic Carbon Priming Effects Under Labile Carbon Inputs

As a potential mediator of soil carbon balance, the PE connects soil carbon input and output, representing a key mechanism for regulating soil carbon dynamics. This is primarily because new substrate inputs provide a greater abundance of nutrients for microbial growth, which in turn leads to microbial assimilation or alterations in life strategies [20]. Microorganisms constitute the primary agents driving organic carbon mineralization [20,62,63]. Microbial community characteristics varied under different glucose addition treatments. In the 0–20 cm soil layer, increased glucose input was associated with elevated levels of ^13^C-PLFAs in each microbial group, while total PLFAs content decreased. This trend likely resulted from the provision of accessible carbon sources from glucose, leading microorganisms to preferentially utilize exogenous glucose rather than soil-derived carbon (Figure 5 and Figure 6). Such a shift may indicate a degree of microbial community restructuring, even though overall biomass remained relatively stable. Biologically, the acquisition of a new carbon source (glucose) may cause microorganisms to allocate more energy and resources toward synthesizing specific lipid molecules (e.g., ^13^C-PLFAs) related to metabolic activity while maintaining a balance in overall biomass for adaptation to the new substrate environment [64]. The findings of this study are consistent with these results. Additionally, the findings of this study indicate that soil MBC, MBN, and PLFAs content for each microbial group and total PLFAs content were highest in the surface layer, exhibiting a decline with increasing soil depth (Figure 6). As soil depth increased, nutrient levels declined in a manner consistent with the observed changes in SOC, total nitrogen, and total phosphorus content. This observation concurs with previous reports of decreasing microbial biomass with soil depth [31], likely due to differences in plant litter and root exudates across soil layers. The accumulation of organic substances, such as litter and humus, at the soil surface provides a rich source of nutrition for the growth and reproduction of microorganisms in the surface layer [65]. With increasing soil depth, nutrient levels decreased in a pattern consistent with changes in SOC, total nitrogen, and total phosphorus content, indicating that nutrient availability plays a critical role in regulating microbial biomass. Moreover, research has demonstrated that plant growth improves the ventilation and hydrothermal conditions of surface soil, thereby offering more favorable living conditions and space for surface microorganisms compared with those at greater depths.

Microbial growth and activity largely depend on substrate availability, which in turn influences the decomposition of soil organic matter through the PE. The results of this study indicate a significant positive correlation between the CPE and CM of the 0–20 cm layer and the ^13^C-G^+^, ^13^C-G^−^, and ^13^C-actinomycetes variables. Among these variables, the addition of exogenous organic matter had a notable impact on the carbon PE, microbial biomass, and PLFAs (Figure 7). After 52 days of incubation, the carbon derived from glucose was primarily assimilated by Gram-negative bacteria (as indicated by 16:1 ω7c and 18:1 ω7c), which can be attributed to their generally high metabolic activity, rapid responsiveness to fresh carbon sources, and overall greater proficiency in utilizing labile organic carbon [66]. The addition of exogenous organic matter provides an additional carbon source for soil microorganisms, which may lead to increased microbial biomass and metabolism. When the abundance or activity of these microbial taxa increases [67], they may accelerate the decomposition of complex organic compounds in SOC through the secretion of specific extracellular enzymes, which may contribute to the mineralization of SOC and the PE [57,68].

PE provides insights into the dynamics of profit and loss as well as the balance of soil nutrient elements to a certain extent. The regulation of soil PE is influenced by the nature and quality of external inputs and the surrounding soil microenvironment. The findings indicate that varying amounts of glucose addition produced positive PE values, exhibiting a distinct upward trend with increasing glucose levels. This linear correlation likely arises from a microbial shift from an energy-limited state to one constrained by nutrients as organic matter inputs increase. The substrate preference and nitrogen mining hypotheses appear to be the primary factors governing the formation and persistence of PE under both low and high organic matter additions [7,69]. Nitrogen can alter the response intensity to exogenous carbon by influencing microbial metabolic strategies. In this study, N content was negatively correlated with the PE in the 0–20 cm and 20–40 cm soil layers but positively correlated with PE in the 40–60 cm layer (Figure 7). Studies indicate that under frequent carbon inputs, K-strategy microorganisms enhance decomposition of decadal SOC to acquire nitrogen (“microbial N mining”). High nitrogen availability may weaken this process; when nitrogen is sufficient, microorganisms do not need to decompose native SOC to obtain nitrogen, thereby reducing their response intensity to exogenous carbon [70]. More directly, research confirms that high nitrogen levels inhibit SOC mineralization, with glucose inputs inducing significantly less carbon release in high-nitrogen soils compared to low-nitrogen soils. This suggests that if surface soil is nitrogen-deficient, its response to exogenous carbon may be stronger; conversely, if nitrogen is sufficient, the response may be weaker than in nitrogen-limited deeper soil [71]. Soil pH can directly influence PE via non-biological mechanisms. A significant positive correlation between soil pH and CPE was observed in the surface layers of soil, whereas deeper layers, which exhibited higher pH values, showed a progressively negative correlation between pH and CPE between pH and CPE (Figure 7). This pattern may be attributed to soils under snowy spruce forests maintaining weakly acidic to alkaline conditions that substantially suppress the activity of acidophilic microorganisms and extracellular enzymes. Conversely, divergent PE outcomes have been reported following the addition of glucose to forest soils with varying pH levels [72]. Specifically, glucose application led to positive PE in neutral soils, whereas acidic forest soils exhibited negative PE. This phenomenon is attributed to the dominance of fungi in acidic soils, where carbon from the decomposition of soil organic matter is primarily allocated to reserve substance synthesis rather than respiration, resulting in a negative PE. Current investigations have emphasized significant variability in PE intensity across various soil environments. An inverse relationship between SOC content and PE intensity under consistent conditions has been reported frequently. A negative correlation between PE intensity and inherent SOC content has been documented [15,16]. Specifically, Mantel testing revealed a markedly negative correlation between SOC and CPE in the 0–20 cm soil layer, a relationship that progressively weakened with increasing soil depth. In the 40–60 cm layer, SOC was positively correlated with CPE; however, as overall organic carbon content declined with depth, PE inhibition was reduced (Figure 7). As SOC content continues to decrease with depth, the negative correlation and PE inhibition also lessen. These observations align with the results of the present study. Mechanistically, soils with low SOC levels exhibit reduced microbial and extracellular enzyme activities. Thus, the addition of external organic matter markedly enhances microbial activity, accelerates the decomposition of soil organic matter, and produces a positive PE [14]. Conversely, in soils with high SOC content, microorganisms may preferentially utilize the carbon and nutrients from the added organic matter, thereby generating a negative PE.

## 5. Conclusions

This study employed glucose as a readily degradable carbon source to investigate how the priming effect responds to varying levels of glucose addition at different soil depths (0–20 cm, 20–40 cm, and 40–60 cm). Glucose addition accelerated SOC mineralization, thereby eliciting a positive PE. The magnitude of this effect varied considerably among soil layers; the topsoil layer (0–20 cm) exhibited the highest cumulative CO_2_ emissions across all sampling locations. While transient inhibition of SOC mineralization was observed in all soil horizons under exogenous C addition, this suppressive effect generally proved ephemeral. Over time, SOC mineralization rates tended to rebound or even enhance, suggesting that the dynamic response mechanism involves nonlinear coupling interactions among soil layer heterogeneity, exogenous organic C input intensity, and time-scale dependency. Analytical results from Mantel test analysis and partial least squares modeling indicated that glucose input exerted the most significant and statistically meaningful positive effect on the CPE of SOC. Both glucose addition and soil depth influence the PE of soil carbon, with associated alterations in the composition and structure of the soil microbial community as well as changes in the soil’s physical and chemical properties. The outcomes of this study provide additional insights into the dynamic transformations within the soil carbon pool, particularly in the framework of climate change impacts on the Tianshan forest ecosystem.

## Data Availability

The original contributions presented in this study are included in the article/Supplementary Material. Further inquiries can be directed to the corresponding author.

## Supplementary Materials

The following supporting information can be downloaded at: https://www.mdpi.com/article/10.3390/microorganisms13081729/s1, Table S1. Background values of physical and chemical properties of sample soils. Table S2. Glucose input amounts under four treatments in different soil layers. Table S3. The physical and chemical properties of the soil measured after 52 days.
