# Supplementary material for: Soil Microbiome Drives Depth-Specific Priming Effects in Picea schrenkiana Forests Following Labile Carbon Input
Source: Microorganisms. 2025 Jul 24;13(8):1729. doi: 10.3390/microorganisms13081729 (PMC12388014; doi:10.3390/microorganisms13081729)
Supplement: Supplementary file 1 [file microorganisms-13-01729-s001.zip › microorganisms-3690590-supplementary.pdf]

Table S1. Background values of physical and chemical properties of sample soils.

Depth	SWC	BD	TP	pH	EC (mS/cm)	SOC (g/kg)	TN (g/kg)	P (g/kg)
0-20 cm	37.42±2.79	0.85±0.16	61.66±0.96	6.44±0.08	223.15±43.63	113.59±12.03	13.76±1.42	1.28±0.35
20-40 cm	16.85±2.99	0.89±0.02	59.85±0.23	6.78±0.04	206.00±1.41	51.01±2.67	7.74±1.14	1.23±0.16
40-60 cm	13.85±1.12	1.08±0.03	58.30±1.30	6.90±0.16	205.00±4.24	45.16±2.85	7.61±1.67	1.19±0.15

Note: SWC, BD, TP, pH, EC, SOC, TN, and P are soil water content, bulk density, total porosity, pH value, electric conductivity, soil organic carbon, total nitrogen, and total phosphorus, respectively. Data are presented as mean ± standard error.

Table S2. Glucose input amounts under four treatments in different soil layers.

Soil depth	Treat	SOC (g/kg)	The amount of glucose added (mg)
0-20 cm	LA	113.59	141.99
	MA	113.59	283.98
	HA	113.59	425.96
	CK	113.59	0.00
20-40 cm	LA	51.01	63.76
	MA	51.01	127.53
	HA	51.01	191.29
	CK	51.01	0.00
40-60 cm	LA	45.16	56.45
	MA	45.16	112.90
	HA	45.16	169.35
	CK	45.16	0.00

Note: CK: control treatment; LA: 1 % SOC glucose-added treatment; MA: 2 % SOC glucose-added treatment; HA: 3 % SOC glucose-added treatment; SOC: soil organic carbon.

Table S3. The physical and chemical properties of the soil measured after 52 days.

Soil depth	Treat	EC (mS/cm)	pH	SOC (g/kg)	TN (g/kg)	P (g/kg)
0-20 cm	CK	341.50±2.12	6.93±0.01	110.38±3.36	13.56±0.5	1.17±0.03
	LA	313.50±12.02	6.87±0.01	100.69±8.74	13.50±0.16	1.23±0.08
	MA	295.00±21.21	6.94±0.04	83.70±5.33	10.09±6.77	1.18±0.09
	HA	260.00±7.07	6.98±0.04	77.37±2.45	13.01±0.18	1.33±0.00
20-40 cm	CK	252.50±10.61	6.98±0.01	40.42±0.56	6.69±1.06	1.13±0.05
	LA	246.00±5.66	7.04±0.02	44.68±0.68	7.77±0.18	1.11±0.01
	MA	251.00±19.80	7.07±0.08	38.43±8.58	7.69±0.47	1.02±0.08
	HA	235.50±13.44	6.98±0.07	41.05±0.66	8.41±1.22	1.04±0.17
40-60 cm	CK	325.50±47.38	7.10±0.08	42.00±2.28	7.06±0.66	1.19±0.01
	LA	244.50±0.71	7.07±0.01	41.06±4.42	7.57±0.32	1.06±0.15
	MA	249.50±3.54	7.06±0.02	40.54±3.05	7.50±0.23	1.18±0.01
	HA	276.00±7.07	6.93±0.07	42.89±1.28	7.57±0.95	1.16±0.17

Note: EC, pH, SOC, TN, and P are electric conductivity, pH value, soil organic carbon, total nitrogen, and total phosphorus, respectively. Data are presented as mean ± standard error.
